# The direct transfer approach for transcellular drug delivery

**DOI:** 10.1080/10717544.2023.2288799

**Published:** 2023-11-30

**Authors:** Yi-Fan Wang, Ze-Fan Shen, Fang-yue Xiang, Heng Wang, Pu Zhang, Qi Zhang

**Affiliations:** a Graduate Department, Bengbu Medical College, Bengbu, Anhui, China; b Urology & Nephrology Center, Department of Urology, Zhejiang Provincial People’s Hospital, Affiliated People’s Hospital, Hangzhou Medical College, Hangzhou, Zhejiang, China; c The Second Clinical Medical College, Zhejiang Chinese Medical University, Hangzhou, Zhejiang, China; d School of Stomatology, Zhejiang Chinese Medical University, Hangzhou, China

**Keywords:** Direct transfer, cell membrane, gap junctions, tunneling nanotubes, nanoparticle

## Abstract

A promising paradigm for drug administration that has garnered increasing attention in recent years is the direct transfer (DT) of nanoparticles for transcellular drug delivery. DT requires direct cell-cell contact and facilitates unidirectional and bidirectional matter exchange between neighboring cells. Consequently, DT enables fast and deep penetration of drugs into the targeted tissues. This comprehensive review discusses the direct transfer concept, which can be delineated into the following three distinct modalities: membrane contact-direct transfer, gap junction-mediated direct transfer (GJ-DT), and tunneling nanotubes-mediated direct transfer (TNTs-DT). Further, the intercellular structures for each modality of direct transfer and their respective merits and demerits are summarized. The review also discusses the recent progress on the drugs or drug delivery systems that could activate DT.

## 1. Introduction

Most advanced techniques for drug delivery, despite their success, only enhance the passive diffusion efficiency of drugs in the extracellular matrix (Liu et al., 2019). Consequently, the tissue-penetrating capacity of drugs is hampered when the permeability of the tissue barrier is reduced in pathological conditions (Ayloo & Gu, 2019; Harilal et al., 2020). Drug delivery systems via a transcellular approach can overcome this bottleneck. Transcellular drug delivery is the process by which a drug crosses the lipid bilayer of the cell membrane between different cells to reach the target site and is not limited to the extracellular matrix. Transcellular drug delivery is activated by either of the following two mechanisms: Transcytosis and direct transfer (DT). Transcytosis is a biological event that carries cargo through several rounds of endocytosis and exocytosis in different batches of cells (Jones & Minshall, 2020). Zhou et al. reviewed drug delivery systems via transcytosis in detail (Zhou et al., 2022). DT represents the most unique mechanism of transcellular drug delivery, and its process requires intercellular connection, involving only intracellular pathways. DT has been demonstrated to provide a highway for transcellular drug delivery deep into tissues (Montero et al., 2011; Egusquiaguirre et al., 2012; Ventola, 2012; Ding et al., 2020). However, there is still a lack of understanding of DT. This review article summarizes the methodology for studying DT, the subtypes of DT, and their roles in supporting transcellular drug delivery.

## 2. Basic principles of direct transfer

### 2.1. What is direct transfer?

**Figure 1.**
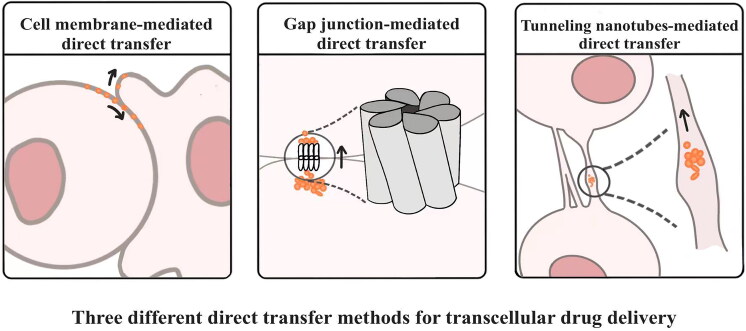
Three different direct transfer methods for transcellular drug delivery (Drawn by us).

Pharmaceutical substances are known to traverse tissues through two distinct pathways: the transcellular and paracellular routes. Nevertheless, drug delivery via the paracellular route adheres to the passive diffusion paradigm, limiting the tissue penetration efficiency (He et al., 2020). In contrast, transcellular transport primarily consists of transcytosis and direct delivery. Transcytosis is an intricate process involving the internalization of cargo into endocytic vesicles, subsequent vesicle trafficking across the cytoplasm, followed by the release of cargo through an exocytic event (Fung et al., 2018). The direct transfer also starts with the cellular internalization of cargo, followed by intracellular trafficking. However, the intercellular transport pathway of direct transfer is only limited to intercellular structures, avoiding drug transport via the extracellular matrix. Drugs can accumulate within cells via direct transfer rather than diffusing the extracellular matrix, resulting in higher intracellular concentration than transcytosis. Moreover, the matter exchange via direct transfer can be more active than transcytosis since the former is independent of exocytosis, which acts as the rate-limiting step during intercellular communication (Zhang et al., 2023). Zhang et al. proved that direct intracellular transfer of the R11-DNA complex induced the delivery of 53.7 ± 11.6% of DNA molecules from the donor cells to the acceptor cells (Zhang et al., 2022). Direct transfer can be further divided according to the intercellular connection into the following: cell membrane-mediated direct transfer, gap junction-mediated direct transfer (GJ-DT), and tunneling nanotubes-mediated direct transfer (TNTs-DT) (Figure 1).

### 2.2. How to identify direct transfer?

It is difficult to distinguish the contribution of transcytosis and DT to intercellular matter exchange during cell co-culture. Specialized models have been employed to reveal the exact transcellular drug delivery efficiency via transcytosis or DT. Typically, ‘infection’ assays among different batches of cells are used to evaluate transcytosis (Zhou et al., 2019). The cells undergo drug treatment followed by thorough rinsing with a pristine medium to eliminate any lingering traces of the drugs. Subsequently, the cells are immersed in a fresh medium to induce exocytosis. The medium, enriched with secretions from the preceding batch, treats the subsequent batch, facilitating the conveyance of secretions without direct transfer. The cargo transportation from donor to acceptor cells primarily occurs intercellularly via transcytosis, assuming the direct cell-cell contact between cell batches is minimal (Zhang et al., 2022).

**Figure 2.**
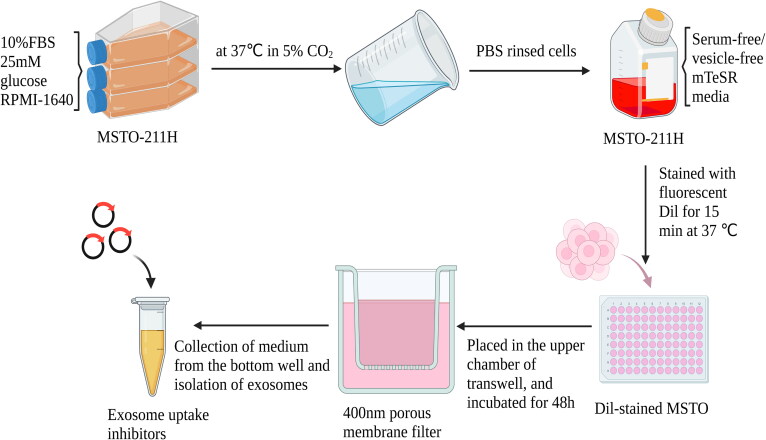
Process for excluding exosomes by transwell analysis (Drawn by us).

Venugopal Thayanithy et al. used a special Transwell assay to exclude the ‘infection’ effect from exosomes while preserving that contributed by TNT-mediated intercellular communication (Thayanithy et al., 2017). The cells were treated with ‘TNT medium (low serum, high glucose medium)’ for 7 days to increase the likelihood of TNT formation. The medium was removed 24 hours before the experiment to reduce interference from exosome secretion; the cells were washed with PBS, and a serum-free medium was added. Next, Dil-labeled cells were loaded in the upper chamber of Transwell inserts with a pore size of 0.4 μm. The cells could not form intercellular gap junctions with cells inside the lower chamber due to the physical barrier of the porous membrane filter. The physical barrier reduced the transport of exosomes by 80%. Moreover, exosome uptake inhibitors such as heparin were applied to inhibit the intercellular transfer of exosomes from the upper chamber to the lower chamber (Atai et al., 2013) (Figure 2).

Krishna C et al. used a Transwell system in 4-mm polyester membrane 6-well plate format to assess intercellular transfer. Mesenchymal stem cells (MSCs) and vascular smooth muscle cells (VSMCs) were placed in the upper and lower chambers, respectively, and co-cultured for 48 hours (Vallabhaneni et al., 2012). They used confocal laser scanning microscopy to observe the intercellular transfer of chloromethylbenzamido, a fluorescent cell tracker dye also known as CM-DiI. The study’s findings revealed that tunneling nanotubes (TNTs) served as a conduit for intercellular exchange between mesenchymal stem cells (MSCs) and vascular smooth muscle cells (VSMCs). The growth of MSCs directed toward VSMCs was hindered after disrupting the TNTs interconnecting MSCs and VSMCs by employing cytochalasin D and latrunculin B (Pasquier et al., 2012).

A universally adopted approach to validate the selectivity of DT-mediated transportation still remains elusive. Generally, DT-mediated transportation is revealed with certainty if the possibility of transcytosis-mediated intercellular transportation can be excluded between donor cells and recipient cells. Different cell types are co-cultured without physical separation to activate the DT-mediated intercellular matter exchange. In this case, the contribution of each modality to DT-mediated transportation was barely distinguishable. Transwell assay is another prevailing co-culture model to test the DT-mediated transportation efficiency. In the Transwell assay, a membrane filter with a pore size of 0.3–0.4 μm was used to ensure its efficacy as an impervious obstacle against cellular migration and specific types of direct cell-cell contact, such as gap junction-mediated direct cell-cell contact. Specific inhibitors can also disrupt specific signaling pathways during cell co-culture, downregulating transcytosis or DT. For instance, Hansen et al. demonstrated that monodansylcadaverine (MDC) and dynasore inhibited the intercellular transfer of α-synuclein by 50% and 91%, respectively (Hansen et al., 2011). The binding site between exosomes and their cell surface receptors, such as heparan sulfate proteoglycans (HSPGs), can be blocked by exosome inhibitors, such as heparin, ammonium chloride, and chloroquine, impede exosome internalization by blocking (Atai et al., 2013). Further, actin polymerase inhibitors cytochalasin D and latrunculin B can disrupt actin polymerization or destabilize actin filaments (Lappalainen et al., 2022), impeding the formation of F-actin-rich tunneling nanotubes (TNT) to inhibit DT. A confocal laser scanning microscope was utilized to analyze the spatial relationship of fluorescent-labeled materials and intercellular structures along with the quantitative analysis of DT-mediated intercellular matter exchange (Giepmans et al., 2006). Particularly, the dynamic transportation of fluorescent-labeled materials through intercellular structures strongly indicated their DT related modality.

## 3. Direct transfer to facilitate the drug delivery

### 3.1. Cell membrane-mediated direct transfer

Cell membrane-mediated direct transfer occurs when two cells are tightly connected, and the cell membranes are crosslinked. In such a situation, proteins and lipids on the cell membrane can be transported along the cell membrane binding site between two neighboring cells. Typically, most lipid transport is likely to occur at membrane contact sites and involves lipid transport proteins (LTPs). LTPS solubilizes lipids and protects them from the aqueous environment during intermembrane transport (Vance, 2015). LTPs act at membrane contact sites (MCSs), regions where two organelles are close. These sites reduce the distance LTPs need to diffuse to transport lipids between membranes, accelerating transport (Reinisch & Prinz, 2021). Niu et al. (2009) reported that cell membrane connections could form and break dynamically during the interactions between adjacent cells, leading to transient fusion of cellular membranes. Further, membrane proteins could migrate to neighboring cells by lateral diffusion during transient membrane fusion. Xu and Ducroux demonstrated that membrane fusion contacts between donor cells expressing HIV envelope (Env) and primary macrophages expressing endogenous HIV receptor CD4 and co-receptors facilitated the intercellular transfer of cyclic GMP-AMP (cGAMP) without disrupting their plasma membrane integrity (Xu et al., 2016).

Trogocytosis is distinctly different from the process where one cell engulfs another larger cell and from endocytosis, where a cell absorbs external substances into vesicles. It involves the direct contact between cells and the active engulfment of parts of the membrane or cytoplasmic contents from the target cell, which are then transferred to the engulfing cell. Trogocytosis is generally activated upon the direct contact between antigen-presenting cells and their target cells but is modulated by other factors, such as their microenvironment, bioactivity, and signaling stimulus (Riond et al., 2007; Miner et al., 2015). Intercellular interactions from immune synapses or intercellular adhesion molecules stabilize the direct cell-cell contact during trogocytosis (Uzana et al., 2012). Later, trogocytosis is triggered in immune cells such as T cells, B cells, and natural killer cells (Riond et al., 2007; Poupot et al., 2008; Doherty et al., 2010; Osborne & Wetzel, 2012; Miner et al., 2015). For example, cytotoxic T-lymphocytes stimulated by antigen-presenting cells (APCs) have been reported to display major histocompatibility class (MHC) I and II molecules inherited from APCs. This is because cytotoxic T-lymphocytes simultaneously and actively acquire the membrane contents of the target cell by translocating lipid fragments from the APC membrane (Miyake & Karasuyama, 2021).

Wang et al. (2022) used targeted farnesylated chemically self-assembled nanorings (f-CSANs) as a biomimetic trogocytosis vehicle to engineer directional cargo transfer between cells. Using hydrophobic interactions, these f-CSANs could be inserted into the cell membrane of donor cells. Flow cytometry was used to quantify the f-CSAN transfer efficiency between Raji and A431-R cells; the CSAN+/mKate + cell population was deemed as the acceptors that received f-CSAN successfully (mKate being a basic red fluorescent protein). After αEGFR-Fn3-Far f-CSAN-modified Raji cells and A431-R cells were mixed and co-cultured for 1 hour, about 80% of the A431-R cells were positively stained with f-CSAN. This experiment demonstrated that the f- CSAN-from Raji cells could efficiently label A431-R cells via trogocytosis. The intercellular transfer efficiency of f-CASN is affected by temperature—At a temperature < 37 °C, 6-fold less f-CSANs were transferred to the acceptor cells (Wang et al., 2022).

Intercellularly localized nanoparticles are either attached to the plasma membrane or embedded in the plasma membrane to form an encapsulation (Tang et al., 2015). When the nanoparticles come into contact with the membrane, the surface of the membrane forms a single connected region. The membrane deforms and wraps around the nanoparticle, creating a membrane-particle contact zone. It is crucial to understand the mechanical behavior of nanoparticles between adjacent cells for membrane-mediated drug transport (Hui et al., 2019). Using molecular dynamics simulations, Wu et al. investigated the intercellular behavior of nanoparticles, including adhesion, encapsulation, and trapping of nanoparticles in a bilayer cell membrane (Wu & Yi, 2021). The nanoparticle-membrane interactions were categorized into dual-membrane capture and single-membrane encapsulation based on the bending stiffness of the membrane, the tension, and the particle size ratio to the intermembrane distance. Completely nonlinear numerical studies have demonstrated that nanoparticles between two membranes with mirrored mechanical properties get trapped between adjacent bilayers at smaller membrane distances. Consequently, they are difficult to internalize by any single cell. The degree of entanglement and attachment energy increases as the membrane-particle contact zone increases, and eventually, the nanoparticle separates from one side of the cell membrane until the membrane patch of the neighboring cell completely captures the particle. The mirror-symmetric system structure loses vertical symmetry for nanoparticles confined between membranes with different mechanical properties. The higher the membrane tension, the smaller the membrane deformation, and the interaction force and entanglement are smaller when the membrane wrapping increases than when the membrane is loose. Under the same conditions, the deformation of the rigid membrane is smaller than a soft membrane to reduce its bending energy cost. Moreover, the interaction force and degree of wrapping of a rigid membrane are also smaller than a comparatively softer membrane. The capability of nanoparticles trapped between bilayers to undergo lateral diffusion might enable them to penetrate solid tumors more effectively. In contrast, the intercellular diffusion of fully wrapped nanoparticles would be significantly reduced. Yue et al. reported the shape-dependent nanoparticle-membrane interactions (Yue et al., 2019). Notably, cylindrical nanoparticles are more likely to be trapped between the cell membranes of two cells than circular nanoparticles.

### 3.2. Gap junction-mediated direct transfer

Intercellular junctions, including gap junctions (GJs), tight junctions (TJs), and adherens junctions (AJs), have been known to be core subcellular components to maintain the association between cells, as well as between the cells and the extracellular matrix (Sinyuk et al., 2018). GJs are considered to play the most crucial role in intercellular communication among all types of intercellular junctions. GJs are specialized protein channels that allow two neighboring cells to communicate directly. The main component is connexin (Cxs). Cx monomers are primarily synthesized in the endoplasmic reticulum, followed by translocation to the Golgi apparatus, where six Cx monomers are assembled into hexameric structures. These structures are transported to the surface of the cell membrane via vesicles, and ultimately, the two half-channels located on different cell membranes form the complete GJ head-to-head manner (Nielsen et al., 2012; Mao et al., 2016). The unique structure of the GJ imparts the property of selectivity for substance transport, i.e. only substances < 1.5 KD are allowed to pass and diffuse freely between two neighboring cells (Bonacquisti & Nguyen, 2019). Such molecules include metabolites, microRNAs, drugs, ions, and other similar cellular metabolites (Brink et al., 2012; Sinyuk et al., 2018; Zhou et al., 2023). However, large molecules, such as most proteins and antibodies, cannot pass through the GJs due to their size. Studies have also shown that mitochondria, microRNA (miRNAs), and some chemotherapeutic agents, such as gemcitabine, can also pass through GJs (Forster et al., 2014; Thuringer et al., 2016; Li et al., 2019). Passive diffusion via GJs does not require energy to drive the transport (Sinyuk et al., 2018). Additionally, the abundance of GJs is deregulated in pathological conditions, such as in tumor tissues, possibly due to altered expression profiles of connexin (Nielsen et al., 2012; Bonacquisti & Nguyen, 2019).

Connexin 43(Cx43) is the most widely expressed protein in GJs. Studies have shown that Cx43 facilitates the direct transfer of drugs and nucleic acids via GJs and bypasses the transcytosis transport pathway to avoid degradation (Korenkova et al., 2020). Soares et al. hypothesized that Cx43 hemichannels at the exosomal membrane allowed the direct transport of substances from exosomes to target cells. Since luciferase can catalyze luciferin to make it glow, a similar biosensing system was used to correlate the efficiency of luciferin transfer from exosomes to cells with the luciferin emission intensity in acceptor cells. The addition of luciferin-loaded exosomes (ExoCx43+ or ExoCx43−) to luciferase-expressing cells (either human embryonic kidney-293Cx43+ or human embryonic kidney-293Cx43−) (HEK-293Cx43+ and HEK-293Cx43−) showed that ExoCx43+ shuttled more luciferin to HEK-293Cx43− compared with ExoCx43-, though the luminescence enhancement was minor. Also, an approximately 40% increase in the luminescence intensity was observed in ExoCx43+- HEK-293Cx43+ cells, compared with ExoCx43+- HEK-293Cx43- cells, suggesting that Cx43 expression in receptor cells could facilitate drug transport (Soares et al., 2015).

Recent studies have also introduced another particular type of cell-derived vesicles, called connectosomes, which contain functional GJ channels and can significantly improve the effectiveness of small molecule chemotherapeutic agents. Connectosomes have been shown to undergo transcellular transport via GJs. A study by Avinash K Gadok et al. showed the intercellular transfer of connectomes. Exposure to calcein red-orange (Decrock et al., 2017) dye-loaded Connectosomes resulted in a 6-fold increase in the average fluorescence of recipient cells, compared with untreated cells. The drug delivery via gap junction could bypass the transmembrane process, indicating that membrane-impermeable drugs could rely on this pathway to improve therapeutic effectiveness (Gadok et al., 2018). A study by Andrea N et al. compared connectomes with commercial liposomes to demonstrate the superiority of drug delivery via GJ. Both connectomes and liposomes were loaded with doxorubicin, respectively, followed by intratumoral injection. Two hours post-injection, the average fluorescence intensity of doxorubicin in the nucleus delivered by connectomes was approximately 7-fold greater than that delivered by liposomes (Trementozzi et al., 2020). Another study by Andrea N et al. demonstrated that fluorescein isothiocyanate (FITC)-conjugated dextran encapsulated in the connectomes was more effectively delivered to the recipient HeLa cells than free FITC-dextran. An increase in the molecular weight of dextran was associated with a significant decrease in intercellular transfer efficiency (Trementozzi et al., 2022).

Liang Zong et al. reported that miRNA could be directly transferred between Hela cells in a connexin-dependent manner via GJs (Zong et al., 2016). Two groups of cells that were transfected with mouse miRNA with GFP and empty non-miRNA construct vector with GFP (NC-GFP), respectively, were co-cultured for 36–48 h to form intercellular gap junctions. Compared with empty vector-transfected and non-co-cultured HeLa cells, the expression of miR-96 and miR-183 in acceptor cells was three-fold higher. The transfer efficiency of miRNA via GJs was regulated by connexin and was calculated to be 29.9 ± 11.9, 10.6 ± 5.47, 7.60 ± 4.80, 11.3 ± 3.33, 0.50 ± 0.16, and 0.24 ± 0.10% in Cx43, Cx26, Cx31, Cx26/30, Cx30, and Cx-null cell lines, respectively.

Li et al. (2021) demonstrated that iron oxide nanoparticles (IONPs) could be transported via GJ, increasing GJs formation between transfected mesenchymal stem cells (MSCs) and C6 glioma tumor cells by upregulating Cx43 levels. Therefore, the sensitivity of tumor cells to suicide genes delivered by MSCs was enhanced. Diverse preparations of the gene complexes, including PEI/pDNA (PEI), PEI/pDNA mixed with magnetosome-like ferrimagnetic iron oxide nanochains (MFIONs) (M + P), and PEI/pDNA/MFIONs complexes (M@P) were used for gene transfection of MSCs, respectively. Cx43 expression was found to be upregulated in M@P and M + P-treated MSCs, while PEI failed to increase Cx43 expression (Li et al., 2021).

GJs are known to play a crucial role in activating anticancer immunity and propagating radiation-induced cell death (Azzam et al., 1998; Zhang et al., 2006) and oxidative stress-induced cell death (Feine et al., 2012; Decrock et al., 2017) to the neighboring cells. This process is known as the bystander effect. GJs transport signals from cells exposed to a specific stimulus to neighboring or more distant cells. The bystander effect in tumor tissue is notable, considering that it facilitates damaged signal transport between adjacent cells. This effect can be exploited to spread chemotherapeutic agents, mostly in interconnected cancer cells, and minimize cytotoxicity to normal tissues. The upregulated expression of Cx43 in cancer cells can increase the permeability of the cancerous physiological barrier to chemotherapeutic drugs (Huang et al., 2001) since Cx43 upregulation is known to activate GJ formation, which serves as an essential pathway in the bystander effect. Based on this principle, Li et al. (2021) further analyzed the experimental data to assess the bystander effects by measuring the viability of C6 glioma cells after co-culturing with MSC-tk (mesenchymal stem cells loaded tumor suicide genes in herpes simplex virus thymidine kinase). The results showed that GFP-C6 glioma cells exhibited the lowest cell viability after being co-cultured with MSCs-tk in 200 μg/mL ganciclovir (GCV), which demonstrated that MFIONs could improve the bystander effect of MSCs-tk on C6 glioma cells and had the potential to improve the sensitivity to suicide genes by upregulating the expression of Cx43 (Li et al., 2021).

### 3.3. Tunneling nanotubes

Initially, Tunneling nanotubes (TNTs) were considered an elongated membrane-like structure, first described by Rustom et al. in 2004 (Rustom et al., 2004). TNTs are long and thin protrusions composed of the cytoskeleton and plasma membrane and connect two adjacent cells (Dubois et al., 2020; Korenkova et al., 2020). They are formed by the *de novo* bridge formation between two protrusions from different cells or the extension of connecting membranes from other cells (Gousset et al., 2009). Both terminals of TNTs are open to the cytoplasm, allowing matter exchange between adjacent cells (Taiarol et al., 2021). TNTs are further divided into two categories: ‘thin TNT’ and ‘thick TNT.’ In thin TNT, the diameter varies between 20 and 700 nm, and in thick TNT, the diameter > 700 nm, and its length can reach several hundreds of micrometers (Huang et al., 2022). ‘Thick’ TNTs are composed of both actin and tubulin filaments, while ‘thin’ TNTs only contain actin filaments. Thus, ‘Thin’ TNTs are more fragile and transient than ‘thick’ TNTs (Veranic et al., 2008; Taiarol et al., 2021), and bidirectional transport of cargo between donor cells and acceptor cells can be achieved only via ‘thick’ TNTs due to the contribution of microtubule-based molecular motors (Sisakhtnezhad & Khosravi, 2015; Taiarol et al., 2021).

TNTs are known to act as intercellular pathways for direct transfer (Table 1). Compared with intercellular junctions, the size of substances via TNTs-mediated direct transfer is wider. For example, TNTs can accommodate ions (Ariazi et al., 2017), neurotransmitters (Pinto et al., 2020), proteins (Scheiblich et al., 2021), nanoparticles (Epperla et al., 2015; Moscariello et al., 2019; Franco et al., 2020), and organelles (Nasoni et al., 2021). The cargo transport via TNTs can bypass the extracellular matrix, and thus, cargo can concentrate within cells, and their penetration into tissues is no longer blocked by barriers in the extracellular matrix (Zhang et al., 2023).

**Table 1.** Delivery systems or nanoparticles that rely on the mechanism of TNT-mediated direct transfer.

Delivery system or nanoparticle	Donor/ Acceptor	Efficiency of direct transfer for acceptor cells	Ref.
Mesoporous silica nanoparticles loaded with doxorubicin	RAW macrophages/ RAW macrophages; RAW macrophages/ HeLa cervical cancer cells; RAW macrophages / A549 lung cancer cells	The positive rate of MSNs’ infection’ in RAW macrophage → A549 cell co-incubation models was 20%, and that in RAW macrophage → Hela cell co-incubation models and in RAW macrophage → RAW macrophage co-incubation models dropped to 14% and 7%.	(Gousset et al., 2009)
Bovine serum /GFP-loaded fluorescent nanodiamonds	HEK293T cells/ HEK293T cells; HEK293T cells/ SH-SY5Y cells; SH-SY5Y cells/ HEK293T cells	The positive rate of BGFNs’ infection’ in HEK293T cell → SH-SY5Y cell co-incubation models was 14%, and that in SH-SY5Y cell→ HEK293T cell co-incubation models were 19%.	(Veranic et al., 2008)
Doxorubicin-loaded ApoEderived peptide and chlorotoxin dually functionalized liposomes	NHAs cells/ NHAs cells→ U87-MG cells/ U87-MG cells→ NHAs cells/ U87-MG cells	The positive rate of AC-Lipo@Dox’ infection’ was 5% (NHA cell → NHA cell co-incubation models), 3.8% (U87-MG cell → NHA cell co-incubation models), and 8.2% (U87-MG cell→ U87-MG cell co-incubation models).	(Sisakhtnezhad & Khosravi, 2015)
M1 macrophages loaded with doxorubicin (termed M1-Dox)	M1macrophages/ SKOV3 tumor cells	The positive rate of internalized doxorubicin in the group ‘doxorubicin-loaded M1 macrophages’ was >85% versus 55% for group ‘free doxorubicin (DoxHCL)’	(Ariazi et al., 2017)
Polyethylene glycol-coated vesicles containing nanodiamond	bEnd.3 cells/ bEnd.3 cells	–	(Scheiblich et al., 2021)

**Figure 3.**
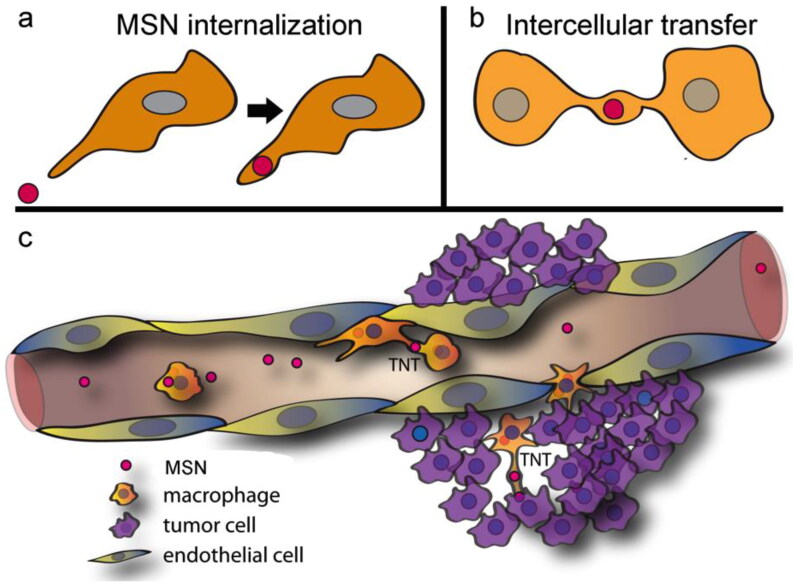
Proposed in vivo trafficking of mesoporous silica nanoparticle (MSN) to the tumor microenvironment. (a) MSN administered intravenously was rapidly internalized by systemic macrophages. (b) Macrophages are highly dynamic and interactive, with intercellular connections, known as tunneling nanotubes (TNT), enabling direct cell-to-cell transfer of MSN to neighboring or distant cells. (c) Proposed movement of MSN to the tumor microenvironment. Reprinted from Franco, S., et al., Direct Transfer of Mesoporous Silica Nanoparticles between Macrophages and Cancer Cells. Cancers, 2020. 12(10). Creative Commons license and disclaimer available from: http://creativecommons.org/licenses/by/4.0/.

Franco et al. (2020) loaded doxorubicin on fluorescently labeled mesoporous silica nanoparticles (MSNs) and transported them via TNTs between homotypic or heterotypic cells, RAW Macrophages to RAW macrophages; RAW Macrophages to HeLa cells; RAW macrophages to A549 lung cancer cells) (Figure 3). The fluorescently labeled nanoparticles were accumulated in large quantities in a small node within the nanotube. Following the completion of direct transfer,24 h post-incubation, the positive rate of MSNs’ infection in RAW macrophage → A549 cell co-incubation models was 20%; RAW macrophage → Hela cell co-incubation models and in RAW macrophage → RAW macrophage co-incubation models dropped to 14% and 7%, respectively (Franco et al., 2020). A. Noureddine et al. reported that the MSNs were primarily transported via thick TNTs between homotypic Hela cells (Noureddine et al., 2021). The positive rate of MSNs’ infection increased with the increase in co-incubation time, ranging from 1.7 to 3.1%.

Epperla et al. (2015) investigated the TNTs-mediated direct transfer of bovine serum/Green fluorescent protein (GFP)-loaded fluorescent nanodiamonds (BGFNs). BGFNs are present in endosomal vesicles within TNTs and could be transported between homotypical human embryonic kidney cells (HEK293T) and bidirectionally transported between HEK293T cells and SH-SY5Y neuroblastoma cells. The positive rate of BGFNs’ infection in HEK293T cell → SH-SY5Y cell co-incubation models was found to be 14%, and that in SH-SY5Y cell HEK293T cell co-incubation models was reported to be 19%. This study indicated the potential of nanodiamonds as a vehicle to shuttle therapeutic proteins deep into tissues.

Formicola et al. (2019) revealed that the TNTs could mediate direct transfer of doxorubicin-loaded ApoEderived peptide and chlorotoxin dually functionalized liposomes (AC-Lipo@Dox) between U87-MG cells themselves, NHAs cells themselves, and U87-MG and NHAs cells. AC-Lipo@Dox preferred ‘thick’ TNTs to support their intercellular transfer. The positive rate of AC-Lipo@Dox’ infection was 5% (NHA cell → NHA cell co-incubation models), 3.8% (U87-MG cell → NHA cell co-incubation models), and 8.2% (U87-MG cell→ U87-MG cell co-incubation models). During the intercellular transfer of AC-Lipo@Dox, ‘thick’ TNTs were formed in 98% of U87 cells, while only 40% of NHA cells contained ‘thick’ TNTs. The difference in TNT numbers between different cells determined the efficiency of intercellular direct transfer of cargo (Formicola et al., 2019).

Although not in the category of nanoparticles. Ling Guo et al. found that doxorubicin-loaded M1 macrophages (M1-Dox) could deliver chemotherapeutic drugs quickly to tumor cells via TNTs from M1macrophages to SKOV3 tumor cells (human ovarian cancer cells) since M1-Dox could sti­mulate ovarian carcinoma cells to form TNTs channels. Free Dox (DoxHCl) was used as a control to demonstrate the high-speed transfer efficiency of M1-Dox. After co-culturing M1macrophages with SKOV3 tumor cells for 30 mins, the % Dox-positive cells and the intense Dox fluorescence were used to examine the kinetics of drug transfer and intracellular accumulation. The positive rate of internalized doxorubicin in the group ‘doxorubicin-loaded M1 macrophages’ was >85% versus 55% for group ‘free doxorubicin (DoxHCL),’ which showed that chemotherapeutic drugs loaded in macrophages were transported via TNTs with better drug delivery efficiency than free chemotherapeutic drugs (Guo et al., 2019). Although these are not nanoparticles, they hold significance for reference purposes.

Pierpaolo Moscariello et al. reported vesicles containing nanodiamonds (Rostami et al., 2017), and the fluorescent NDs were encapsulated by a customized human serum albumin-based biopolymer (polyethylene glycol) coating (dcHSA-PEG), called dcHSA-NDs transferred between bEnd.3 cells (A type of endothelial cell) via TNTs. The dcHSA-NDs-vesicles were also found to be transferred between primary astrocytes and primary neurons. However, the study did not mention the efficiency of NPs transfer between cells (Moscariello et al., 2019).

Organelles are also known to be transported between cells via TNTs. TNTs facilitate the transport of mitochondria, endosomes, and lysosomes into the target cells (Gerdes & Carvalho, 2008; Marzo et al., 2012; Abounit et al., 2016; Sáenz-de-Santa-María et al., 2017; Burt et al., 2019; Pinto et al., 2021). Aneta Dydowiczová et al. reported the bidirectional transport of mitochondria via TNTs. The direction of mitochondria trafficking within TNTs varied based on cell types and exogenous stimulus. For example, mitochondria-contained vesicles were transported unidirectionally between neuronal cells (Onfelt et al., 1950, 2006) but bidirectionally among macrophages (Liang et al., 2021). Recent TNTs were reported to allow bi-directional translocation between cells (Domhan et al., 2011; Lim & Tang, 2012). With the lysosomal trafficking in TNTs, prion-like proteins in lysosomes, such as PrP^Sc^, α-synuclein, tau, polyQ aggregates, and Aβ assemblies, have been shown to spread to neighboring cells to induce neurodegenerative disease (Rostami et al., 2017; Victoria & Zurzolo, 2017). Rostami et al. found that aggregated alpha-synuclein (α-SYN) was transported by TNTs in a prion-like manner between human astrocytes (Rostami et al., 2017). Specific organelle-targeting drugs have the potential to ride their counterpart organelles to promote the efficiency of direct transfer via TNTs since organelles also act as drug delivery systems (Li et al., 2021),.

## 4. Conclusions and perspectives

Direct transfer acts as an alternative mechanism for drugs to achieve intercellular transport. Direct transfer can be further classified into cell membrane-mediated direct transfer, gap junction-mediated direct transfer, and tunneling nanotube-mediated direct transfer. Since drug delivery via direct transfer does not involve passive diffusion in the extracellular space, it has higher efficiency than that via other transcellular transport mechanisms. Despite these transport phenomena, the effectiveness of drug delivery to deep tissues is limited by several factors.

The size and characteristics of molecules: Many drugs are relatively large molecules that might not effectively pass through cell membranes, gap junctions, or TNTs (Sinyuk et al., 2018; Zhang et al., 2021). These channels have size limitations and may not be able to accommodate larger drug molecules, impeding their transport between cells.

Cellular barriers: Drugs are typically known to traverse multiple cellular and extracellular barriers in deep tissues. These barriers include the extracellular matrix, cell membranes, etc.

Differential expression: Gap junctions and TNTs are not uniformly distributed across cell types and tissues. Their presence and function are known to vary significantly between organs and regions (Monaghan et al., 1996; Han & Wang, 2021). This differential expression can affect the ability of drugs to utilize these transport mechanisms for deep tissue penetration. Researchers and drug developers continue to study strategies to enhance deep tissue delivery and effectively use intercellular transport mechanisms, including designing nanoparticles to carry drugs and promoting drug penetration by modifying the size, charge, and surface properties of the nanoparticles. Another approach involved the use of cell-penetrating peptides (CPPs) rich in arginine to deliver impermeable drugs (Sun et al., 2022).

However, the activity of direct transfer is highly dependent on the biological process, which might be known to be different in different types of cells or under different stimuli. For example, the formation of GJs is generally inhibited in cancerous tissue, especially in those with a high risk of metastasis; thus, GJ-mediated direct transfer might be invalid. Another concern involves the biosafety of drug delivery systems, which rely on direct transfer. The mechanism of direct transfer might drive drugs to escape into normal tissues, inducing the off-target effect (Keelan et al., 2015; Li et al., 2019). Additionally, there exists a lack of understanding of the molecular target to facilitate interaction with the drug delivery systems (Zhu et al., 2021). Further investigations are critical to reveal the mechanisms behind the phenomenon of direct cell-to-cell transfer.

## Data Availability

All data is contained within the manuscript.
